# East meets West: current issues relevant to integrating Chinese medicine

**DOI:** 10.1186/1749-8546-7-20

**Published:** 2012-09-03

**Authors:** Emily Yen Wong, Barak Gaster, Sum Ping Lee

**Affiliations:** 1 Li Ka Shing Faculty of Medicine, University of Hong Kong, 6/F, William MW Mong Block, 21 Sassoon Road, Pokfulam, Hong Kong SAR, China; 2 Department of Medicine, School of Medicine, University of Washington, 1959 NE Pacific St, Seattle, Washington, 98195, USA; 3 University of Hong Kong 3/F Ap Lei Chau Clinic, 161 Main Street Ap Lei Chau, Hong Kong, China

## Abstract

This article describes the challenges that integration of Chinese medicine (CM) and biomedicine are likely to bring for improving safety, research, education, and cross-disciplinary communication. Potential strategies to meet these challenges are suggested, including the use of accessible language for the Western biomedical community, and further development of whole-system randomized controlled trials that support individualized treatment approaches.

## Introduction

Chinese medicine (CM) practices, such as herbal medicine and acupuncture, are well known and established in clinical practice and research. This article takes a balanced descriptive approach to highlighting the opportunities and challenges that the integration of CM with Western medicine may bring.

CM is a traditional medicine grounded in empirical observations that has developed over thousands of years. Acupuncture is perhaps the best known and studied CM treatment modality. Other common modalities include Chinese herbal medicines, moxibustion, and *tui na* therapeutic massage. In Western countries including the United States, CM is categorized under complementary and alternative medicine (CAM), and considered distinct from conventional biomedical approaches to treatment.

CM has roots going back more than 2,000 years. Ancient medical texts have been refined and interpreted extensively over the millennia, giving rise to multiple, sometimes competing, branches of study and practice. The term “Traditional Chinese Medicine” refers to the modern standardized approach mandated by the Chinese government in the 1950s [1]. The goal was to preserve CM, which was perceived to be endangered at the time. Systematic terminology and formal academic training programs were developed to enhance the science of the field, and simultaneously certain mystical elements were purged.

### CM in China

Since the integration of CM and Western medicine in mainland China in the 1950s, physicians in China have been cross-trained substantially in both disciplines. Western biomedical doctors are required to receive a significant proportion of CM training as part of their core medical education and many often acquire additional training [1]. Many CM schools devote at least one-third of the curriculum to Western medicine, and most have further shifted toward Western medicine in recent years [1,2]. As a result, many doctors in China are facile in the use of both Western medicine and CM in daily practice, sometimes utilizing both modalities, depending on the patient’s preference or condition. For example, patients with neck pain may receive diagnostic X-rays, and then treatment with a combination of CM (*e.g.* acupuncture) and Western medicine (*e.g.* nonsteroidal anti-inflammatory drugs) [2]. Such integration of clinical practices is common in China, but is still considered unconventional in Western countries.

The collaborative integration between CM and Western medicine works not just at the level of individual practitioners, but also at the institutional level. According to official statistics, 2,688 (13.6%) of 19,712 hospitals in China were specifically designated as CM specialty hospitals [3]. However, the vast majority of Western medicine hospitals in China offer CM services, and virtually all CM hospitals provide Western medicine services, including high-tech diagnostic testing and modern surgical techniques [1,2]. At the health clinic level, approximately half of clinicians practice Western medicine, one-third practice a blend of Western medicine and CM, and the remainder practice only CM [1]. CM dispensaries contribute almost half of the pharmaceutical revenues for Western medicine hospitals in China, while Western medicine drugs comprise a large proportion of the pharmacy sales at CM hospitals [4]. The strong revenue potential of herbal sales and high demand for CM services enable many hospitals to remain solvent in the highly competitive healthcare market, where individual hospitals must secure out-of-pocket patient revenues to sustain clinical business operations.

CM herbal medicines are widely embraced by the public for both disease treatment and prevention, enjoying robust demand from domestic and international markets and strong support from the Chinese government. As China’s population ages, the burden of disability and chronic disease grows. CM is crucial to government health policies, which seek to achieve high levels of cost-effectiveness and patient satisfaction [5].

Although public and private payers in China provide broad coverage for CM services, the third-party payer system is piecemeal at best for medical services. Most patients still cover a high proportion of expenses out-of-pocket, even in urban medical centers [6]. Some studies have demonstrated that, for rehabilitation purposes, acupuncture is quite affordable compared with Western medicine practices, such as physical therapy [7]. However, patients without financial means or government-based insurance have limited access to the hybrid of CM and Western medicine services favored by more affluent families [7]. While patient satisfaction has not been widely studied in China, some authors have reported high levels of satisfaction when patients have the choice to access both CM and Western medicine services [8].

### CM in the West

Various forms of CM are currently practiced in over 120 countries worldwide [9]. These overseas versions of CM, *e.g.* Western acupuncture, may have developed derivative techniques or concepts. According to a recent review, CM publications outside China has risen rapidly over the past two decades in a wide range of languages, including English, Japanese, French, Korean, and German [10].

The export of Chinese herbal medicines to the West has grown to over US$500 million per year for herbal pharmaceuticals, primarily to the US and European markets [11]. CM is highly prevalent in the United States [12], and is especially widespread among ethnic Chinese immigrants in countries such as the United Kingdom [13]. Popular English-language textbooks and biomedical literature on CM have been published, reporting on major European research, which is often supported by government health authorities.

The regulation of CM practitioners and herbal medicines by governments has generally been slow to develop, and typically only occurs in response to specific safety concerns. Significant progress has primarily been made in practitioner licensing and accrediting of educational programs. Integrative models of care are emerging in the West to combine complementary therapeutic approaches such as CM with conventional approaches [14]. High satisfaction rates with CAM modalities, such as CM, are generally associated with holistic approaches that promote patient autonomy and satisfaction [15].

## Discussion

While rigorous research into the mechanisms and efficacy of acupuncture has continued to accumulate in recent years [16,17], a number of critical pragmatic and cultural issues have also emerged.

(1) Safety The safety of acupuncture has been widely accepted since the universal adoption of disposable needles and clean needle techniques. Serious adverse events, such as pneumothorax, are extremely rare [18]. In addition to its strong safety record, acupuncture has been shown to be cost-effective in treating back pain and migraine headaches [19,20].

In contrast to acupuncture, CM herbal toxicity remains a major safety concern for CM. Some CM botanicals are toxic when used in excess [21], while others have been found to be contaminated with heavy metals or adulterated with Western medicine pharmaceutical products [22]. Some quality assurance standards have been established for products and equipment in some areas, but globally the herbal medicine industry has largely been allowed to regulate itself [23]. Safety risks are thus taking a backseat to economic pressures in driving good manufacturing practices.

(2) Communication CM diagnostics and therapeutics remain obscure to most Western medicine practitioners, partly because of different terminology and concepts. Although important interpretative and translational work has been in progress, a great deal remains to be done [24]. Developing a modern language for traditional concepts would be a significant challenge; however, this also represents a great opportunity to make CM more accessible to Western audiences.

CM theory is still based on classics such as the *Huangdi Nei Jing*, also known as the *Yellow Emperor’s Inner Canon*, that date back more than 2,000 years. Current CM has largely been adapted to modern clinical practices, but the language of CM therapies and practice has barely changed. CM modernization should encourage the gradual introduction of a modern language for scientific inquiry and analysis, while still preserving the role of classics in empirical observations for developing fundamental principles of CM. Such a dynamic transition would actually perpetuate the process for updating, interpreting, and refining CM over the millennia [25].

The theory behind CM, such as *qi* and meridians, presents special challenges to the integration of Eastern and Western medicine, given that these concepts are not recognized in the West. With the use of clear explanatory metaphors, the communication barriers can be overcome. For example, *qi* can be described as “life energy that flows along channels in the body” [24]. Western clinicians are thus encouraged to accept a pluralistic paradigm of how the human body might function, which may bring about a more robust understanding of what constitutes health. Medical education in China has been conducted in just such a pluralistic fashion for decades, and thus its population benefits from modern innovations as well as traditional therapies.

CM and Western medicine share common goals to improve the health of the public, including functional outcomes of sustainable and accessible care. Effective communication for future collaborative efforts will require recognition that there is more to gain from thoughtful, informed, and open-minded cooperation.

(3) Research Western medicine will face challenges in seeking innovative models for studying CM. Research on CM modalities does not fall easily into the evidence-based medicine paradigm, with its strong reliance on randomized controlled trials [26]. However, specific approaches to research design have been suggested and validated with regard to acupuncture [27]. Research on CM herbal therapies adds further substantial complexities, but ongoing work on the standardization of such studies and reporting protocols will likely bear fruit in the near future [28].

Further work is needed to determine better methodologies for investigation into individualized treatments, because CM emphasizes customized therapeutic approaches, which are often highly tailored to the individual. Whole-system research takes a more effective approach to studying therapies in a real-world environment, allowing individualized treatment plans [29,30]. Such novel research models have the potential to invigorate the scientific underpinnings of CM, thereby allowing a deeper understanding of the mechanisms by which CM might work.

(4) Education The Institute of Medicine in the United States has recommended that all US physicians should be trained to “competently advise” their patients regarding CAM therapies, such as CM [31]. At present, many medical schools in the United States address this mandate by teaching students to inquire about CAM practices, but standardizing education for providing advice on such therapies remains difficult.

Innovative educational approaches that are based on evidence and utilize a common lexicon would greatly facilitate medical training and foster improved collaboration between the East and the West. Such educational innovations should emphasize inter-practitioner communication and standardized measures of quality and safety to support a common platform for safe and reliable clinical cooperation, similar to efforts that are already underway between other health professional fields.

## Conclusion

The integration of CM and Western medicine represents a potentially mutually beneficial therapeutic partnership. Better regulation of traditional CM herbs, improved translation of classics into modern language, further development of whole-system methods for randomized controlled trial designs, and better interdisciplinary approaches to education are on the horizon. An informed approach to health, science, and economic policies is needed to help mitigate the potentially negative influences brought about by purely market-driven approaches.

## Competing interests

There are no competing interests for any of the participating authors.

## Authors’ contributions

EYW drafted the manuscript and worked with SPL on approach and structure of this commentary. BG revised and edited the manuscript and contributed additional material before initial submission. EYW responded to reviewers and revised the manuscript. All authors read and approved the final manuscript.
